# Editorial: Glomerular filtration rate in Chronic Kidney Disease

**DOI:** 10.3389/fmed.2022.1105384

**Published:** 2023-01-10

**Authors:** Ana Maria Cusumano, Guillermo Rosa Diez, Carmen Tzanno-Martins

**Affiliations:** ^1^Centro de Educación Médica e Investigaciones Clínicas Norberto Quirno (CEMIC), Buenos Aires, Argentina; ^2^Posgraduate Department, Italian Hospital of Buenos Aires University Institute, Buenos Aires, Argentina; ^3^Brazilian Society of Nephrology, São Paulo, Brazil

**Keywords:** CKD, GFR, glifozins, vitamin D, acidosis, uric acid

**Chronic Kidney Disease** (CKD) is defined as “abnormalities of kidney structure or function, present for more than 3 months, with health implications” and is classified according to the cause, the glomerular filtration rate (GFR) category, and the magnitude of albuminuria (1). So, the diagnosis of CKD progression is based on two key parameters: GFR and the presence and extent of albuminuria. GFR is considered the best global index of renal function since its decrease usually correlates with functional renal mass. GFR can be easily estimated (eGFR) through equations that include endogenous analytes such as creatinine or cystatin C, alone or combined, and anthropometric and demographic factors. At the individual level, the accuracy of an eGFR equation is defined as (p30), which means that around 85% of GFR determinations are within ±30% of mGFR. eGFR should not be used when extreme body composition is present, such as patients with anorexia nervosa, cirrhosis, debilitated elderly, severe obesity, or when there is a need to administer nephrotoxic drugs with a narrow therapeutic option. When necessary, mGFR can be measured using radioisotopes or contrast media.

The understanding that a reliable and consistent GFR estimation (which means reproducibility under the same conditions) is central for the practice of nephrology in particular and medicine in general. Taking into account its limitations, at present, eGFR is not only a powerful tool for identifying CKD, but it has become fundamental for physicians for early detection, clinical diagnosis, monitoring of progression, indication for admission to replacement therapy, calculation of the dose of drugs excreted by the kidney, and in preparation for invasive diagnostic or therapeutic procedures. As an epidemiological tool, eGFR is not only a simple method to estimate the global burden of CKD, but an instrument for identifying risk factors for progression, understanding the epidemiology of kidney disease concerning different social groups (particularly vulnerable ones), and establishing public policies that intend to reduce CKD at the population level. In kidney disease investigation, it is necessary to determine the risks and benefits of new drugs over CKD progression.

This volume includes several updates on different aspects of GFR in CKD. The evolution from the physiologic concept of glomerular filtration until its measurement through GFR and its present use as a clinical and epidemiological health tool; to estimate GFR in different populations and clinical situations, such as in pediatrics, older adults, and kidney donors and in obese patients and individuals born with low birth weight, all are detailed in several manuscripts. There is also a good update on different equations for eGFR at different pediatric and adult ages.

It is known, at least since the beginning of the century, that CKD increases the risk of cardiovascular disease and vice versa, as both diseases not only share some cardiovascular risk factors (2) but because each one is a risk factor for the other. The complex variety of mechanisms leading from vascular injury to CKD and from CKD to vascular injury are described, in particular the two-way path between arterial stiffness and renal dysfunction. About living kidney donors, there is little consensus and no evidence about what is considered an appropriate GFR for accepting someone as a kidney donor. mGFR, though the gold standard, is not available everywhere. A combination of two methods is suggested (for example, creatinine clearance and eGFR).

The volume also includes several research articles.

Once again, it was confirmed that metabolic acidosis, defined as a serum bicarbonate level < 22 mmol/l is an independent risk factor for kidney progression.

Serum uromodulin (sUmod), a biomarker of tubular mass and kidney function, in patients referred for coronary angiography showed a linear increase in all-cause and CV mortality, from the group with high sUmod-high eGFR **<** Low sUmod-High eGFR **<** High sUmod-low GFR **<** low sUmod-low eGFR was found. The conclusion was that sUmod additionally to creatinine or cystatin C enables a more precise risk modeling for all-cause and CV mortality.

Using data from the NANES 2017-2018, the interactive impacts of smoking (a modifiable risk factor) and sleep on kidney function were studied in a cross-sectional study; the results showed that normal sleep duration was a protective and more crucial factor for non-smokers than for smokers.

The study about uric acid and impairment of renal function in non-diabetic hypertensive patients concluded a uric acid level >7.5 mg/dl is a probable optimal cutoff value for predicting kidney function deterioration.

In older adults with CKD followed longitudinally, the patent of deterioration of GFR and malnourishment predicted mortality and kidney failure.

Finally, two manuscripts focused on the novel effects of two drugs in CKD: Sodium-Glucose Cotransporter 2 inhibitors (SGLT2i) or gliflozins, and vitamin D. About the SGLT2i, the different proposed mechanisms of action which would result in improving cardiac and renal outcomes are described. Briefly: SGLT2i inhibits renal glucose reabsorption at the proximal tubules by blocking the SGLT2 cotransporters; the resulting glycosuria reduces hyperglycemia and improves Hb_A1c_. The simultaneous increase in sodium excretion reverses the tubule-glomerular feedback, reducing the mechanism of damage of glomerular hyperfiltration and slowing the progression of CKD. Calorie loss from glycosuria results in weight loss, increased insulin sensitivity, enhanced lipid metabolism, and probably lessened lipotoxicity. Metabolism moves toward gluconeogenesis and ketogenesis, two probably protective effects for the heart and the kidneys.

The manuscript Non-classical Vitamin D Actions for Renal Protection aimed to update on CKD-induced alterations in both systemic and local bioactivation of vitamin D and calcitriol actions, and to develop strategies to effectively reduce the progression of CKD, without considering the extent of secondary hyperparathyroidism but attenuating its most potent inducers: systemic inflammation, arterial hypertension, and renal and CV damage. The pathophysiology that causes the reduction mediated by calcitriol/VDR (vitamin D receptor) in the proinflammatory and hypertensive signals not related to the decrease in klotho was analyzed.

Some conclusions emerge from this volume:

- GFR continues to be a powerful tool for the diagnosis and follow-up of CKD patients and as an epidemiology tool.
- Physicians should be alert about clinical situations where persistent hyperfiltration is present as a mechanism of damage (low birth weight, kidney donors, aging, obesity, CKD, etc.).
- Scopes and limitations of eGFR must be known, so as to identify when mGFR should be preferred.
- Kidney function deterioration increases the risk of CV-related death in CKD and other non-communicable chronic diseases.
- New drugs, like gliflozins, will contribute to reducing both the progression of CV disease and CKD, added to angiotensin-converting enzyme (ACE) inhibitors or angiotensin II receptor antagonists.

What can we expect about eGFR in the near future? Probably GFR estimation should advance to population-type specific equations, with the race component resolved. In obesity stages II and III, the research could conclude in original formulas for estimating GFR and resolve the doubt about standardizing or not to body surface area. Finally, the recent description of the shrunken pore syndrome, diagnosed by a ratio eGFRcystatin C/eGFRcreatinine < 0.60 (3), which can happen without proteinuria, reinforces applying both formulas. Future CKD guidelines probably will delineate when the simultaneous use of both equations is needed.

## Author contributions

All authors listed have made a substantial, direct, and intellectual contribution to the work and approved it for publication.
